# A Predictive Nomogram of In‐Hospital Mortality After 48 h for Atrial Fibrillation Patients in the Coronary Care Unit

**DOI:** 10.1002/clc.70017

**Published:** 2024-09-17

**Authors:** Wenhui Wang, Linlin Liu, Lu Jin, Bo Hu

**Affiliations:** ^1^ Department of Cardiology, Shanghai East Hospital, School of Medicine Tongji University Shanghai China; ^2^ Department of Cardiology Seventh People's Hospital of Shanghai University of Traditional Chinese Medicine Shanghai China; ^3^ Department of Cardiology Anda Hospital Shanghai China

**Keywords:** atrial fibrillation, coronary care unit, mortality, prediction model

## Abstract

**Background:**

Patients with atrial fibrillation (AF) suffer a higher risk of death, and it is necessary to develop prediction tools for mortality risk in critically ill patients with AF. This study aimed to develop a novel predictive nomogram of in‐hospital mortality after 48 h in the coronary care unit (CCU) for patients with AF.

**Methods:**

We collected information on CCU patients with AF from the “Medical Information Mart for Intensive Care‐III” database and developed a nomogram model for predicting the all‐cause mortality risk after 48 h in the hospital. Key variables were selected by univariate logistic and least absolute shrinkage and selection operator regression. The independent predictors with *p* < 0.05 were screened out by multivariate logistic regression. A predictive nomogram was constructed using these independent predictors, and the model calibration and discrimination were evaluated.

**Results:**

This study finally enrolled 1248 CCU patients with AF, and the in‐hospital mortality was 17% (209/1248). The predictive nomogram was constructed by 13 selected independent predictors, including age, smoking status, acute kidney injury, chronic obstructive pulmonary disease, ventricular arrhythmia, shock, urea, red cell distribution width, leucocytosis, continuous renal replacement therapy, continuous positive airway pressure, anticoagulation, and heart rate. The area under the curve of the nomogram was 0.803 (95% confidence interval 0.771–0.835). The nomogram was verified to have good accuracy and calibration.

**Conclusions:**

This study developed a novel nomogram containing age, acute kidney injury, and heart rate that can be a good predictor of potential in‐hospital mortality after 48 h in CCU patients with AF.

## 1 Introduction

Atrial fibrillation (AF) is a common disease with high morbidity and mortality and often coexists with several chronic diseases, especially in coronary care unit (CCU) patients [1]. The Framingham Heart Study found that patients with AF experienced higher risks of death with a 1.5‐ to 1.9‐fold hazard ratio than those without AF [2]. Moreover, critically ill patients with new‐onset AF experienced a higher in‐hospital mortality rate [3]. Additionally, the incidence of AF increases with age and will be a significant epidemiological and public health challenge in an aging society [4].

The commonly used clinical risk scores of AF (i.e., CHADS2, CHA2DS 2‐VASc, HAS‐BLED score) are used for the risk assessment of stroke and bleeding. They have poor predictive power for mortality, with an area under the curve (AUC) below 0.65 [5]. Adding other risk factors to these scores slightly improved the predictive power [6]. Other risk scores, such as the BASIC‐AF score, require electrocardiography (ECG) and echocardiography, and the ABC score requires the inclusion of uncommon biomarkers, limiting their clinical application [7, 8]. Otherwise, in critically ill patients, the acute physiology and chronic health assessment (APACHE II) and sequential organ failure assessment (SOFA) scores correlate with disease severity and mortality [9]. However, their utility in critically ill patients with AF is uncertain. It is necessary to further develop a prediction model for the mortality risk in AF patients, especially in severe AF patients, and this prediction model combines the advantages of high prediction efficiency and simplicity.

The nomogram is a visualization of a clinical prediction or a prognostic model. It has good clinical value as a predictive tool that addresses the clinical needs of specific populations [10]. Critical AF patients in the CCU are at high risk of death, and a practical prediction model is needed [11]. This study aimed to develop a novel nomogram to predict all‐cause in‐hospital mortality for AF patients in the CCU after 48 h.

## 2 Materials and Methods

### 2.1 Data Access

Using the Medical Information Mart for Intensive Care‐III database, we collected information on CCU patients with AF from 2001 to 2012. The study population was AF patients with a CCU stay ≥ 48 h since admission, and the AF was either pre‐existing or new‐onset during hospitalization. The outcome was all‐cause in‐hospital mortality after 48 h. The establishment of the database is approved by the Institutional Review Boards of the Massachusetts Institute of Technology and the Beth Israel Deaconess Medical Center. We have passed the “Protecting Human Research Participants” exam and obtained permission to access the data set (code: 43259734). The original data of the database were obtained with informed consent, and the database encrypted the information. Therefore, this study waived informed consent and complied with the Declaration of Helsinki.

Information such as the basic admission information, laboratory indicators within 48 h of admission, comorbidities, medications, interventions, and SOFA and APACHE II scores were extracted. As part of the admission information, sex, age, body mass index (BMI), smoking status, heart rate (HR), and survival status were recorded. Among the laboratory indicators were the baseline white blood cell count, hemoglobin, hematocrit (HCT), red cell distribution width (RDW), platelet count, partial thromboplastin time (PTT), serum potassium (K^+^), anion gap, bicarbonate (HCO_3_^−^), urea, serum creatinine (Scr) and glucose (Glu) levels, as well as Scr and Glu retested within 48 h. Comorbidities included chronic obstructive pulmonary disease (COPD), cerebral infarction, shock, chronic kidney disease (CKD), diabetes mellitus (DM), hyperlipidaemia, hypertension (HT), acute coronary syndrome (ACS), prior myocardial infarction (priorMI), heart failure (HF), and ventricular arrhythmia. The medications were recorded such as angiotensin‐converting enzyme inhibitors (ACEIs), beta‐blockers, statins, diuretics, antiplatelet drugs, and anticoagulation drugs (mostly warfarin). The interventions included continuous renal replacement therapy (CRRT), continuous positive airway pressure (CPAP), and reperfusion therapy. Furthermore, the variables with missing values of less than 20% were imputed using multivariate imputation. In addition, HR was classified as < 80 beats per minute (bpm) (as reference), 80–110 bpm, and > 110 bpm according to the controlled HR [12]. Obesity was defined as BMI ≥ 30 (kg/m^2^). Leucocytosis was defined as white blood cell count > 10*10^9^/L. Anemia was defined as hemoglobin < 10 g/dL. Thrombocytopenia was defined as platelet count < 100*10^9^/L. Acute kidney injury (AKI) was defined as an increase of Scr within 48 h by ≥ 0.3 mg/dl or ≥ 1.5 times baseline [13]. Blood glucose variability (GV) was obtained as the standard deviation/mean*100% of the Glu levels within 48 h.

### 2.2 Inclusion and Exclusion Criteria

The criteria for inclusion in the database were: (1) admission to the CCU; (2) CCU stay ≥ 48 h; and (3) a discharge diagnosis of AF with the following ECG findings: irregular R–R intervals, no discernible P waves, and lasting at least 30 s [14]. AF has the International Classification of Diseases‐Ninth Revision code 427.31.

The exclusion criteria were: (1) pregnant or with malignant diseases; (2) < 18 or > 90 years; and (3) records of the noninitial CCU admissions in the patients with repeated admissions.

### 2.3 Statistical Analysis

The enrolled patients were separated into the survivor group and nonsurvivor group according to in‐hospital mortality, and their hospitalization information included continuous and categorical variables. The continuous variables in this study were found to be nonnormally distributed using the Shapiro‒Wilk method. They were expressed as the median (interquartile range), and analyzed by the Wilcoxon rank‐sum. On the other hand, categorical variables were expressed as the number of cases and percentage of the total sample size [*n* (%)] and analyzed by Fisher's exact test.

Then, the variables with *p* < 0.1 in the univariate logistic regression were put into the least absolute shrinkage and selection operator (LASSO) regression model. Potential mediating variables were restricted into the model, and they could be identified by clinical experience. The variables with nonzero coefficients in the LASSO model were identified as the key variables. The independent predictors with *p* < 0.05 were screened out from these key variables using multivariate logistic regression (backward stepwise procedure) [15]. A nomogram was constructed using these independent predictors to predict the in‐hospital mortality of CCU patients with AF. Then, the Hosmer‒Lemeshow (H‒L) test and calibration plot were used to calibrate the nomogram. The efficacy of the nomogram, SOFA score, and APACHE II score was evaluated by receiver operating characteristic (ROC) curves and decision curve analysis (DCA), comparing the nomogram's AUC with the two scores by the DeLong test. The bootstrap method of resampling 1000 times was used for internal validation.

All statistical tests were performed by SPSS Statistics for Windows (version 26.0; IBM Corporation, Armonk, New York, United States) and R software (version 4.0.3; R Core Team, Vienna, Austria), with a two‐sided *p* < 0.05 defined as statistically significant.

## 3 Results

### 3.1 Baseline Characteristics

This study enrolled 1248 CCU patients with AF (Figure S1). The median age was 76 (ranging from 31 to 89) years old, and 511 (41%) were female. The median hospital stay was 4 days. Among them, 209 patients (17%) died in‐hospital (nonsurvivor group), and the remaining 1039 patients consisted of the survivor group. The baseline characteristics are shown in Table 1 (Table 1).

**Table 1:** Baseline characteristics of the enrolled patients with AF.

Variables	Survivor group (*n* = 1039)	Nonsurvivor group (*n* = 209)	*p*
Age, years	75 (66, 81)	78 (69, 83)	0.021
Female, *n* (%)	429 (41)	82 (39)	0.581
Obesity, *n* (%)	342 (33)	53 (25)	0.032
Smokers, *n* (%)	182 (18)	21 (10)	0.008
Heart rate, *n* (%)			< 0.001
< 80 bpm	466 (45)	60 (29)	
80–110 bpm	439 (42)	103 (49)	
> 110 bpm	134 (13)	46 (22)	
SOFA	4 (2, 6)	6 (4, 9)	< 0.001
APACHE II	44 (34, 54)	58 (47, 71)	< 0.001
Laboratory indicators			
Leucocytosis, *n* (%)	529 (51)	135 (65)	< 0.001
Anemia, *n* (%)	250 (24)	41 (20)	0.166
Thrombocytopenia, *n* (%)	47 (5)	8 (4)	0.655
HCT, %	33.9 (30.2, 38.1)	34.4 (30.7, 37.4)	0.394
RDW, %	15 (14, 16)	15 (14, 17)	< 0.001
PTT, s	35.4 (29.3, 50.2)	37.1 (29.8, 57.4)	0.108
K^+^, mmol/L	4.2 (3.8, 4.6)	4.3 (3.9, 4.9)	0.015
Anion gap, mmol/L	14 (12, 17)	16 (14, 19)	< 0.001
HCO_3_^‐^, mmol/L	25 (22, 28)	23 (20, 27)	< 0.001
Scr, mg/dL	1.2 (0.9, 1.8)	1.4 (1.0, 2.0)	0.001
Urea, mg/dL	26 (17, 42)	34 (23, 53)	< 0.001
Glu, mmol/L	130 (105, 168)	137 (108, 195)	0.041
GV, %	23.8 (16.7, 31.8)	26.7 (19.8, 38.0)	< 0.001
Comorbidities, *n* (%)			
CKD	185 (18)	35 (17)	0.714
AKI	305 (29)	103 (49)	< 0.001
COPD	34 (3)	13 (6)	0.041
HBP	414 (40)	66 (32)	0.025
DM	352 (34)	73 (35)	0.770
Hyperlipidaemia	260 (25)	42 (20)	0.129
HF	663 (64)	143 (68)	0.204
priorMI	93 (9)	22 (11)	0.472
ACS	165 (16)	46 (22)	0.031
Shock	130 (13)	67 (32)	< 0.001
Cerebral infarction	16 (2)	3 (1)	0.910
Ventricular arrhythmia	139 (13)	49 (23)	< 0.001
Medications, *n* (%)			
ACEIs	574 (55)	69 (33)	< 0.001
Statins	573 (55)	99 (47)	0.040
Diuretic	843 (81)	154 (74)	0.014
Beta‐blocker	895 (86)	152 (73)	< 0.001
Anticoagulant drugs	629 (61)	66 (32)	< 0.001
Antiplatelet drugs	826 (80)	148 (71)	0.006
Interventions, *n* (%)			
CRRT	15 (1)	20 (10)	< 0.001
CPAP	180 (17)	69(33)	< 0.001
Reperfusion	182 (18)	43 (21)	0.294

*Note:* Continuous data were presented as median (interquartile range), and categorical data were presented as *n* (%).

Abbreviations: ACEIs, angiotensin‐converting enzyme inhibitors; ACS, acute coronary syndrome; AF, atrial fibrillation; AKI, acute kidney injury; APACHE II, acute physiology and chronic health assessment II; bpm, beats per minute; CKD, chronic kidney disease; COPD, chronic obstructive pulmonary disease; CPAP, continuous positive airway pressure; CRRT, continuous renal replacement therapy; DM, diabetes mellitus. Glu, blood glucose; GV, glucose variability; HBP, hypertension; HCO_3‐_, bicarbonate; HCT, hematocrit; HF, heart failure; K^+^, serum potassium; n, number of cases; priorMI, prior myocardial infarction; PTT, partial thromboplastin time; RDW, red cell distribution width; Scr, serum creatinine; SOFA, sequential organ failure assessment.

The nonsurvivor group had more elderly patients (*p* = 0.021), worse HR control (*p* < 0.001), higher SOFA and APACHE II scores (both *p* < 0.001), and were less likely to have obesity or a smoking status (*p* = 0.032 and *p* = 0.008, respectively) than the survivor group, and the two groups did not differ significantly in sex (*p* > 0.05).

The laboratory indicators showed that the nonsurvivor group had a higher proportion of leucocytosis, higher levels of admission RDW, K^+^, anion gap, Scr, urea, Glu, and GV, and lower HCO_3‐_ than the survivor group (all *p* < 0.05). Moreover, the nonsurvivor group had a higher proportion of comorbidities, including AKI, COPD, HBP, ACS, shock, and ventricular arrhythmia, than the survivor group (all *p* < 0.05). In contrast, the nonsurvivor group had lower proportions of patients taking anticoagulant drugs, ACEIs, statins, diuretics, beta‐blockers, and antiplatelet drugs (all *p* < 0.05). In terms of interventions, the nonsurvivor group had a higher proportion of CRRT and CPAP (both *p* < 0.001), with no significant difference in the proportion of patients given reperfusion therapy (*p* = 0.294).

### 3.2 Screening of Independent Predictors

**Figure 1:**
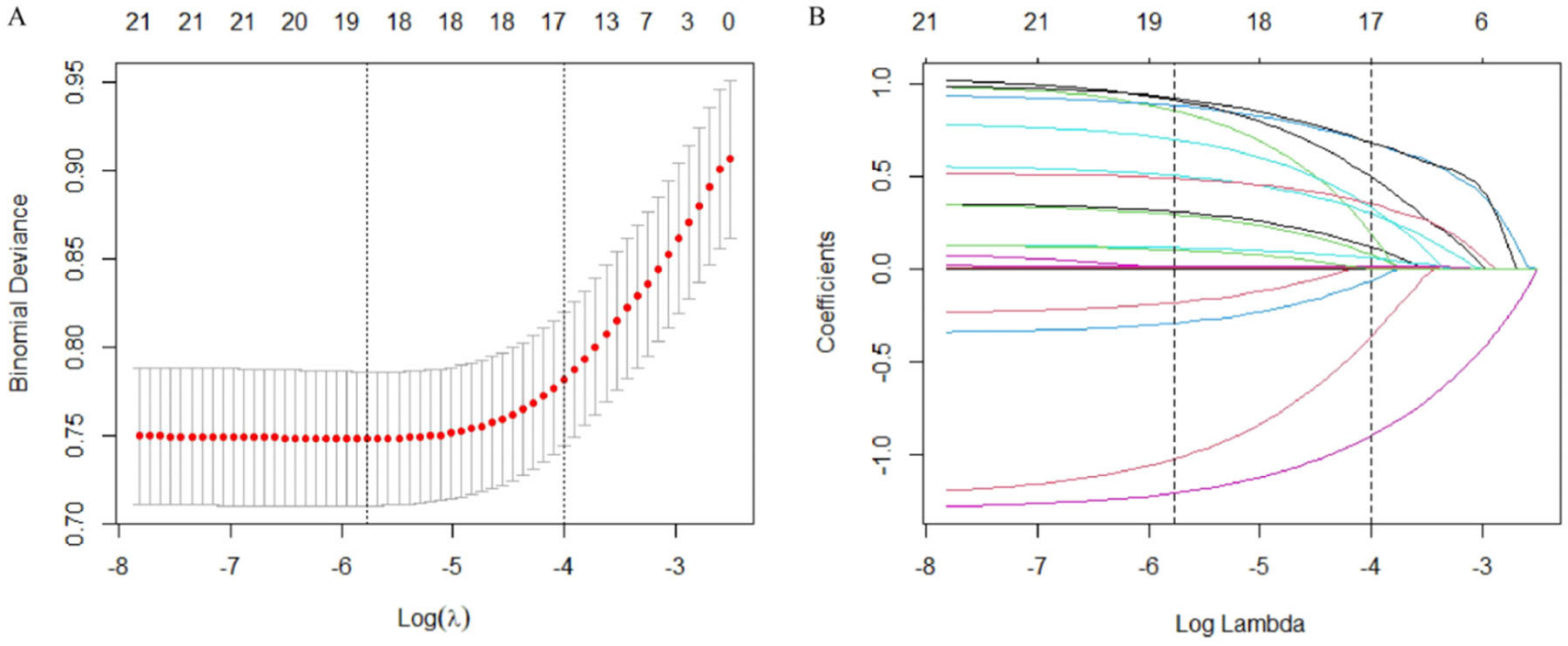
Selection of the key variables that predicted in‐hospital mortality after 48 h in CCU patients with AF using LASSO regression. (A) Cross‐validation diagram of the LASSO regression model. The two dashed lines represent the best lambda selection range, and the lambda value that minimized the binomial deviance of the model was selected. (B) Coefficient profile diagram of the LASSO model, showing the change in the coefficients of 21 variables as log (lambda) increased. Variables whose coefficients became zero were eliminated from the model. AF, atrial fibrillation; CCU, coronary care unit; LASSO, least absolute shrinkage and selection operator.

Of the variables with *p* < 0.1 that are shown in Table 1, medications (other than anticoagulant drugs) were excluded from the next step of the LASSO model. Because medications (other than anticoagulant drugs) were the consequence of clinical treatment for concomitant diseases in AF patients, they were considered potential mediating variables. The LASSO regression model selected 19 key variables (Figure 1). These key variables were used to construct a multivariate logistic regression model, and 13 independent predictors with *p* < 0.05 were screened out (Table 2).

**Table 2:** The independent predictive factors of in‐hospital mortality identified by univariate and multivariate Logistic regressions.

Variables	Univariate			Multivariate		
	OR	95%CI	*P*	OR	95%CI	*P*
Age, years	1.02	1.00–1.03	0.021	1.02	1.00‐1.04	0.017
Heart rate, *n* (%)						
< 80 bpm	Ref	Ref	Ref	Ref	Ref	Ref
80–110 bpm	1.82	1.29–2.57	0.001	1.64	1.11–2.43	0.013
> 110 bpm	2.67	1.74–4.10	< 0.001	3.15	1.94–5.13	< 0.001
Smokers, *n* (%)	0.53	0.33–0.85	0.008	0.30	0.17–0.55	< 0.001
Shock, *n* (%)	3.30	2.3–4.65	< 0.001	2.64	1.75–3.97	< 0.001
Ventricular arrhythmia, *n* (%)	1.98	1.37–2.86	< 0.001	2.20	1.43–3.37	< 0.001
Anticoagulant drugs, *n* (%)	0.30	0.22–0.41	< 0.001	0.27	0.19–0.38	< 0.001
CPAP, *n* (%)	2.35	1.69–3.27	< 0.001	2.73	1.78–4.17	< 0.001
AKI, *n* (%)	2.34	1.73–3.16	< 0.001	1.77	1.25–2.51	0.001
RDW, %	1.13	1.05–1.21	0.001	1.14	1.05–1.24	0.003
Urea, mg/dL	1.01	1.00–1.02	0.001	1.01	1.00–1.02	0.008
COPD, *n* (%)	1.96	1.02–3.78	0.045	2.76	1.30–5.89	0.008
CRRT, *n* (%)	7.22	3.63–14.36	< 0.001	2.73	1.17–6.40	0.021
Leucocytosis, *n* (%)	1.76	1.29–2.39	< 0.001	1.45	1.02–2.07	0.041
Obesity, *n* (%)	0.69	0.49–0.97	0.033	0.69	0.47–1.02	0.065
Anion gap, mmol/L	1.10	1.06–1.14	< 0.001			
GV, %	1.02	1.01–1.03	< 0.001			
K^+^, mmol/L	1.26	1.05–1.51	0.014			
HBP, *n* (%)	0.70	0.51–0.96	0.025			

Abbreviations: AKI, acute kidney injury; bpm, beats per minute; CI, confidence interval; COPD, chronic obstructive pulmonary disease; CPAP, continuous positive airway pressure; CRRT, continuous renal replacement therapy; GV, glucose variability; HBP ‐ hypertension; K^+^, serum potassium; *n*, number; OR, odds ratio; RDW, red cell distribution width; Ref, control group.

### 3.3 Construction and Validation of the Nomogram

**Figure 2:**
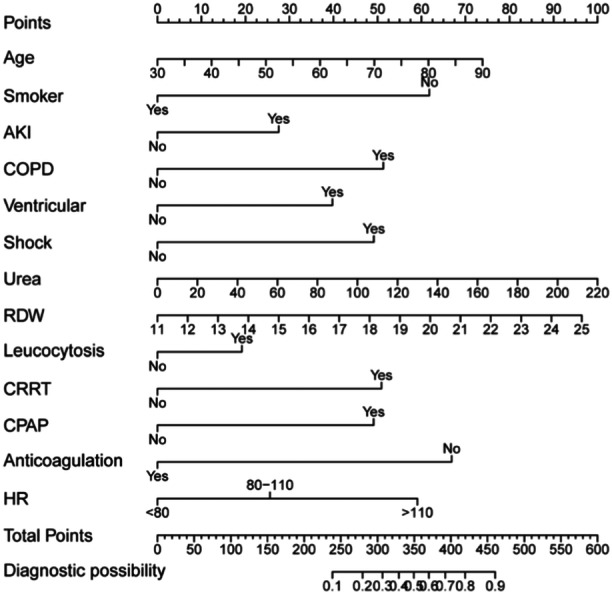
Nomogram for predicting in‐hospital mortality after 48 h in CCU patients with AF. How to use: Each predictor has a specific value for an AF patient. The point is projected onto the first row to obtain the corresponding score of each predictor (i.e., “ventricular arrhythmia = Yes” = 40 points). The points of all predictors are added together to obtain the total points. The risk of in‐hospital mortality of AF patient can be obtained by projecting the total points to the probability in the last row. AF, atrial fibrillation; CCU, coronary care unit.

**Figure 3:**
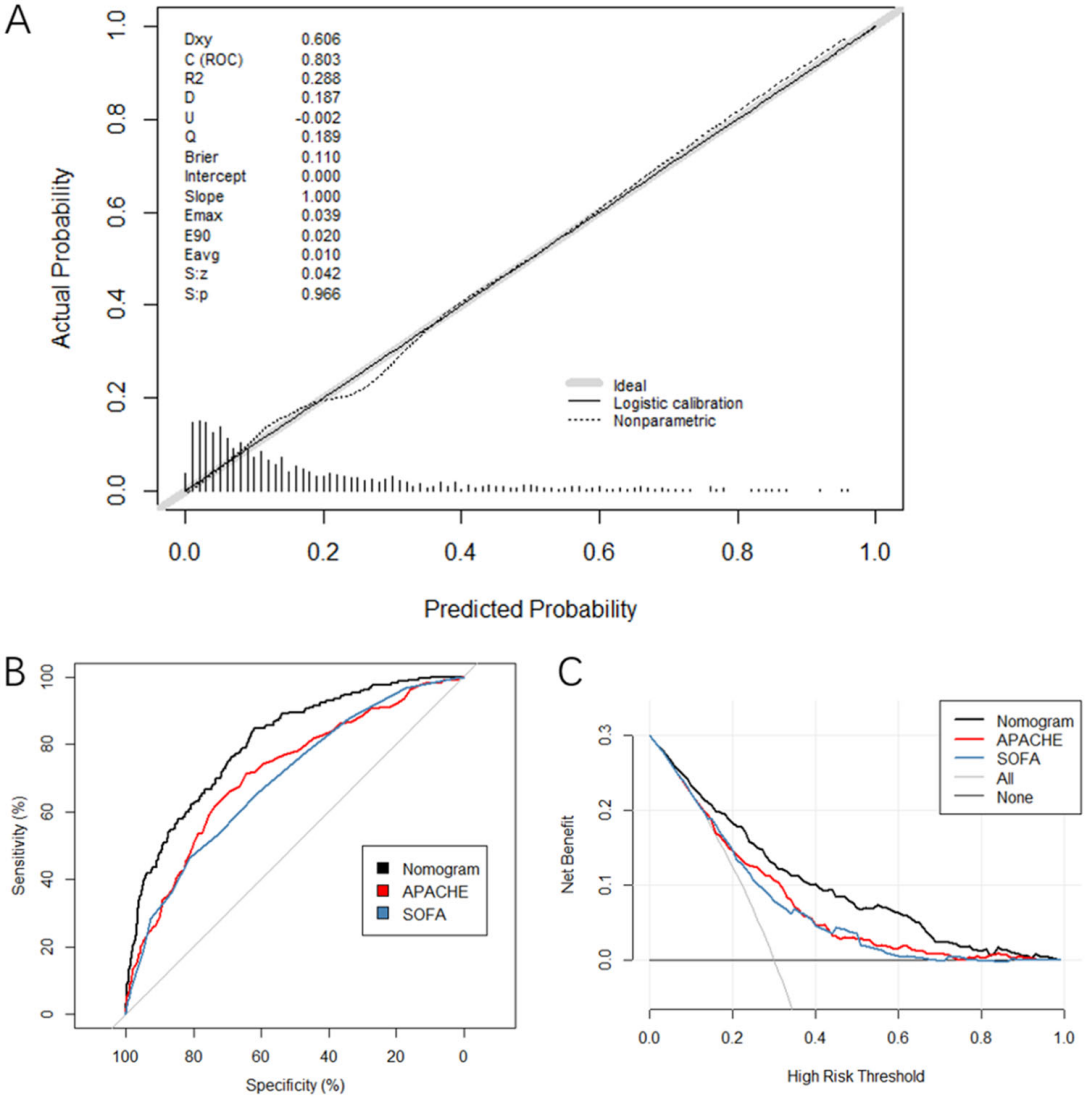
The calibration and predictability of the predictive nomogram in this study. (A) Calibration curves of the predictive nomogram, SOFA score and APACHE II score. The *x*‐axis represents the predicted risk of bleeding. The *y*‐axis represents the actual diagnosed bleeding risk. The diagonal dotted line represents a perfect prediction by an ideal model. The solid line represents the performance of the nomogram, where a closer fit to the diagonal dotted line represents better prediction efficiency. (B) ROC curves of the predictive nomogram, SOFA score, and APACHE II score. The AUC of the predictive nomogram was higher than the SOFA score (80.3% vs. 69.7%, *p* < 0.001) and the APACHE II score (80.3% vs. 71.7%, *p* < 0.001). C. DCA curves of the nomogram, SOFA score, and APACHE II score. The *x*‐axis represents the threshold probability, and the *y*‐axis represents the net benefit. The gray line represents the assumption that all patients had in‐hospital death events. The black line represents the assumption that no patients had in‐hospital death events.

A nomogram was constructed to predict in‐hospital mortality using these 13 independent predictors, including age, smoking status, AKI, COPD, ventricular arrhythmia, shock, urea, RDW, leucocytosis, CRRT, CPAP, anticoagulation, and HR (Figure 2). The H–L test suggested an *X*‐squared of 4.17 (*p* = 0.841) for the nomogram, and the calibration curve showed that the predicted probabilities of the nomogram fitted the observed probabilities satisfactorily (Figure 3A). The internal validation suggested that the accuracy was 0.84, indicating good validation of the nomogram model.

### 3.4 The Predictive Ability of the Nomogram Compared With the SOFA and APACHE II Scores

The ROC curves of the predictive nomogram, SOFA score, and APACHE II score in this study were plotted (Figure 3B). The AUC of the nomogram was 0.803 (95% confidence interval 0.771–0.835), and it was higher than that of the SOFA score (0.803 vs. 0.697, *p* < 0.001) and the APACHE II score (0.803 vs. 0.717, *p* < 0.001). The DCA curves of the nomogram, SOFA, and APACHE II scores showed that the nomogram had a higher clinical benefit than the other two scores (Figure 3C).

SOFA, sequential organ failure assessment; APACHE II, acute physiology and chronic health assessment II; ROC, receiver operating characteristic; DCA, decision curve analysis; AUC, area under the curve.

## 4 Discussion

According to this study, the in‐hospital mortality for patients with AF who were admitted to the CCU and stayed for more than 48 h was 17% (209/1248). Thirteen independent predictors of in‐hospital mortality were finally identified based on the LASSO and logistic regression, which comprehensively covered basic admission information, laboratory indicators, comorbidities, medications, and interventions of AF patients (Figure 2). Moreover, a nomogram with the above predictors was constructed to predict in‐hospital mortality after 48 h in CCU patients with AF. This is the first predictive model of in‐hospital mortality after 48 h for AF patients in the CCU. The model has demonstrated a promising predictive power, outperforming the SOFA and APACHE II scores.

AF patients have a prolonged duration of mechanical ventilation and intensive care and, more importantly, higher mortality [16, 17]. Medical staff in the CCU often encounter patients with comorbid AF without a competent method to assess the risk. One of the reasons is the wide variation in the major admission diseases of CCU patients, while AF is often a concomitant disease. It can be seen that the SOFA score and APACHE II score, which apply to critically ill patients, did not give a satisfactory risk assessment of AF patients in the CCU (AUC = 0.697 and 0.717, respectively). On the other hand, some scores require the inclusion of the heart ventricular conduction time obtained from an ECG and echocardiographic data, which dramatically increases the difficulty of completing the assessment [7]. The CCU team craves a highly accurate and easily accessible risk assessment system.

The population of this study was AF patients in the CCU who were hospitalized for cardiac reasons. By a 27‐year temporal‐trend observation of mortality in patients with cerebral hemorrhage, it was found that there was no significant change in mortality within 48 h, while the mortality from 48 h to 30 days decreased with time [18]. Reassessment of disease severity after 48 h of admission in critically ill patients can better predict mortality [19]. The study of predictors of mortality after 48 h of hospitalization in critically ill AF patients will help to identify higher‐risk groups and improve overall treatment efficacy.

The traditional AF scores of stroke risk and bleeding risk are recommended for treatment strategy development; they have a moderate predictive accuracy and are not recommended for mortality risk prediction [12, 20]. The SOFA and APACHE II scores also showed only moderate predictive accuracy in this study. Meanwhile, the nomogram presented in this study had excellent predictive efficacy with an AUC higher than 0.80 and high calibration and accuracy. Moreover, this nomogram had good clinical applicability because the predictors involved were all standard information. In addition, this study innovatively included some dynamically monitored indicators in the multivariate regression analysis and found that AKI was one of the independent risk predictors for AF patients, while glycaemic variability was not. Short‐term deterioration of renal function was independently correlated with high mortality in critically ill patients with AF [21]. This finding, while improving the prediction accuracy of the nomogram, reminds the CCU medical staff of the need for enhanced monitoring of renal function to reduce the rate of missed AKI [22]. However, we should also recognize that this is a preliminary model, and further external validation is needed.

In the final nomogram, we observed that anticoagulant drugs and smoking were correlated with lower in‐hospital mortality in CCU patients with AF. Anticoagulant drugs were an independent protective factor in AF patients, which was consistent with previous findings [23]. However, the result of smoking was contrary to our usual perception, and this “smoker's paradox” has also repeatedly appeared in observational studies of patients with myocardial infarction and stroke [24, 25]. In the present study, only the 13 variables that were most strongly associated with the outcome were included in the nomogram, and other confounding factors were not included. Detailed information on smoking history was not collected. This corrected residual survival advantage may have resulted from not including enough covariates [26]. After all, our study focused on the predictive ability of the nomogram in patients with AF. Smoking was associated with poor outcomes in cardiac patients during long‐term follow‐up [27].

Beyond smoke, the association between the other variables included in the model and the risk of mortality seems pretty straightforward [28]. This does not apply to COPD. AF patients with comorbid COPD are predicted to have a higher risk of death, which has been supported by retrospective and prospective studies [29]. Comorbid COPD and CKD contribute most to all‐cause mortality of AF patients after long‐term follow‐up [30]. However, the pathological mechanisms of epidemiological association between COPD and AF are still lurking beneath the surface [31]. One reason may be that the hypoxia, hypercapnia, and electrolyte imbalance caused by COPD are detrimental to patient health [29]. On the other hand, COPD is associated with worsening AF [32]. The pulmonary hyperinflation of COPD brings about an irreversible enlargement of the right atrium with more impaired RA function; the concomitant hypoxia of COPD shortens the atrial effective expiration period (AERP) and exacerbates autonomic dysfunction; the inflammatory cytokines secreted by recurrent acute exacerbations of COPD are also associated with the progression of AF [31, 33, 34]. In the rabbit model of COPD, the atrial complex exhibits higher electrical remodeling and fibrosis [35]. These may be potential reasons for the association observed between COPD, AF, and mortality.

## 5 Limitations

The first limitation was that there might be subject to selection bias since it was a single‐center retrospective study and the most severe patients (such as those who did not reach the CCU) were excluded by design. Second, some potential predictive variables were not included in this study due to a percentage of missing values well above 20%, and these variables included plasma troponin levels, and N‐terminal brain natriuretic peptide levels [36]. The current nomogram has shown excellent predictive ability; adding the variables mentioned above may further improve the model's predictive performance. Therefore, the current findings could be further verified in a larger population and multiple centers.

## 6 Conclusions

This study successfully developed a novel predictive nomogram of in‐hospital mortality risk after 48 h for AF patients in the CCU. The nomogram contained 13 standard variables including age, smoking status, AKI, COPD, ventricular arrhythmia, shock, urea, RDW, leucocytosis, CRRT, CPAP, anticoagulation, and HR. It better predicts the potential in‐hospital mortality after 48 h in CCU patients with AF than the SOFA and APACHE II scores.

## Ethics Statement

The establishment of the Medical Information Mart for Intensive Care III database is approved by the Institutional Review Boards (IRB) of the Massachusetts Institute of Technology (MIT, Cambridge, MA, USA) and the Beth Israel Deaconess Medical Center (BIDMC). We have passed the “Protecting Human Research Participants” exam and obtained permission to access the data set (authorization code: 43259734). The original data of the database had obtained the informed consent and the database encrypted the information. Therefore, this study waived informed consent and complied with the Declaration of Helsinki.

## Conflicts of Interest

The authors declare no conflicts of interest.

## Supporting information

Supporting information.

## Data Availability

The data supporting this study are available from Medical Information Mart for Intensive Care III database. The data that support the findings of this study are available from the corresponding author upon reasonable request.
