# Supplementary figures and images for: A Predictive Nomogram of In‐Hospital Mortality After 48 h for Atrial Fibrillation Patients in the Coronary Care Unit
Source: Clin Cardiol. 2024 Sep 17;47(9):e70017. doi: 10.1002/clc.70017 (PMC11408711; doi:10.1002/clc.70017)

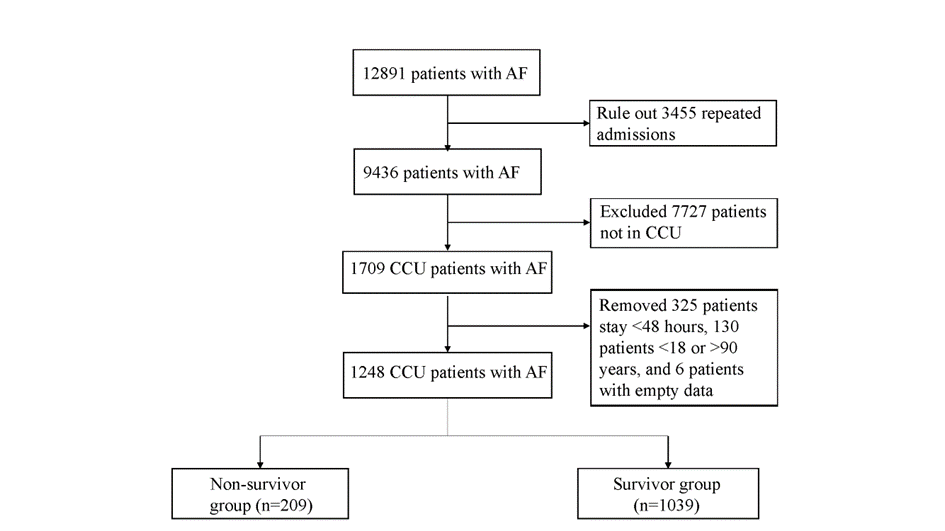


**Supplementary **Figure 1** Flow chart of the patients enrolled in the study**

**AF, atrial fibrillation.**

Supplement: Supplementary file 1 — Supporting information. [file CLC-47-e70017-s001.docx]
